# Direct statistical inference for finite Markov jump processes via the matrix exponential

**DOI:** 10.1007/s00180-021-01102-6

**Published:** 2021-04-19

**Authors:** Chris Sherlock

**Affiliations:** Department of Mathematics and Statistics, Lancaster University, Lancaster, UK

**Keywords:** Markov jump process, Likelihood inference, Bayesian inference, Matrix exponential

## Abstract

Given noisy, partial observations of a time-homogeneous, finite-statespace Markov chain, conceptually simple, direct statistical inference is available, in theory, via its rate matrix, or infinitesimal generator, ${\mathsf {Q}}$, since $\exp ({\mathsf {Q}}t)$ is the transition matrix over time *t*. However, perhaps because of inadequate tools for matrix exponentiation in programming languages commonly used amongst statisticians or a belief that the necessary calculations are prohibitively expensive, statistical inference for continuous-time Markov chains with a large but finite state space is typically conducted via particle MCMC or other relatively complex inference schemes. When, as in many applications ${\mathsf {Q}}$ arises from a reaction network, it is usually sparse. We describe variations on known algorithms which allow fast, robust and accurate evaluation of the product of a non-negative vector with the exponential of a large, sparse rate matrix. Our implementation uses relatively recently developed, efficient, linear algebra tools that take advantage of such sparsity. We demonstrate the straightforward statistical application of the key algorithm on a model for the mixing of two alleles in a population and on the Susceptible-Infectious-Removed epidemic model.

## Introduction

A *reaction network* is a stochastic model for the joint evolution of one or more populations of *species*. These species may be chemical or biological species (e.g. Wilkinson 2012), animal species (e.g. Drovandi and McCutchan 2016), interacting groups of individuals at various stages of a disease (e.g.Andersson and Britton 2000), or counts of sub-populations of alleles (e.g. Moran 1958), for example. The state of the system is encapsulated by the number of each species that is present, and the system evolves via a set of *reactions*: Poisson processes whose rates depend on the current state.

Typically, partial and/or noisy observations of the state are available at a set of time points, and statistical interest lies in inference on the unknown rate parameters, the filtering estimate of the state of the system after the latest observation or prediction of the future evolution of the system. The usual method of choice for exact inference on discretely observed Markov jump processes (MJPs) on a finite or countably infinite state space is Bayesian inference via particle Markov chain Monte Carlo (particle MCMC, Andrieu et al. (2010)) using a bootstrap particle filter (e.g. Andrieu et al. (2009); Golightly and Wilkinson (2011); Wilkinson (2012); McKinley et al. (2014); Owen et al. (2015); Koblents and Miguez (2015)). Other MCMC and SMC-based techniques are available e.g. Kypraios et al. (2017), and, a further latent-variable-based MCMC method when the statespace is finite Rao and Teh (2013).

Particle MCMC and SMC, however, are relatively complex algorithms, even more so when a bootstrap particle filter (simulation from the process itself) is not suitable and a bridge simulator is necessary, such as when observation noise is small or when there is considerable variability in the state from one observation to the next; see Golightly and Wilkinson (2015), Golightly and Sherlock (2019), Black (2019). In cases where the number of states, *d*, is finite, direct exact likelihood-based inference is available via the exponential of the infinitesimal generator for the continuous-time Markov chain, or rate matrix, ${\mathsf {Q}}$. Whilst such inference is conceptually straightforward, it has often been avoided in practice for general MJPs, except in cases where the number of states is very small e.g. Amoros et al. (2019). The computational cost of each iteration of particle MCMC is proportional to the number of particles used and, for efficient estimation; see Doucet et al. (2015),Sherlock et al. (2015) this is approximately linear in the size of the statespace, *d*. In contrast, Matrix exponentiation has a computational cost of ${\mathcal {O}}(d^3)$, which, together with a lack of suitable tools in R, could explain the lack of uptake of this method. However, conceptually simple statistical inference via the matrix exponential is entirely practical in many cases even when the number of states is in the thousands or higher, and it has been used successfully in a subclass of these situations ( e.g. Jenkinson and Goutsias (2012), see Sect. 2.2). There are three main reasons why this is possible:

1. Matrix exponentials themselves are never needed; only the *product* of a vector and a matrix exponential is ever required.
2. The matrices to be exponentiated are infinitesimal generators and, as such, have a *special structure*; furthermore, the vector that pre-multiplies the matrix exponential is non-negative.
3. The matrices to be exponentiated are usually *sparse*; tools for basic operations with large, sparse matrices in C++ and interfacing the resulting code with R have recently become widely available; see Eddelbuettel and Sanderson (2014), Sanderson and Curtin (2018).

The sparsity of ${\mathsf {Q}}$ arises because the number of possible ‘next’ states given the current state is bounded by the number of reactions, which is typically small. This article describes matrix exponential algorithms suitable for statistical application in many cases, and demonstrates their use for inference, filtering and prediction. Associated code provides easy-to-use R interfaces to C++ implementations of the algorithms, which are typically simpler and often faster than more generally applicable algorithms for matrix exponentiation.

Section 1.1 describes the Susceptible-Infectious-Removed (SIR) model for the evolution of an infectious disease and the Moran model for the mixing of two alleles in a population, then briefly mentions many more such models where the statespace is finite, and a few where it is countably infinite. The two main examples will be used to benchmark and illustrate the techniques in this article. As well as being directly of use for models with finite state spaces, exponentials of finite rate matrices can also be used to perform inference on Markov jump processes with a countably infinite statespace; see Georgoulas et al. (2017) and Sherlock and Golightly (2019). The latter uses the uniformisation and scaling and squaring algorithms as described in this article, while the former uses the less efficient but more general algorithm of Al-Mohy and Higham (2011) (see Sect. 3).

Section 2 of this article presents the likelihood for discretely and partially observed data on a finite-statespace continuous-time Markov chain and presents two ‘tricks’ specific to epidemic models, that allow for a massive reduction in the size of the generators that are needed compared with the size of the statespace. Section 3 describes the Matrix exponential algorithms and Sect. 4 benchmarks some of the algorithms and demonstrates their use for inference, filtering and prediction. The article concludes in Sect. 5 with a discussion.

### Examples and motivation

Both by way of motivation and because we shall use them later to illustrate our method, we now present two examples of continuous-time Markov processes, where a finite, sparse rate matrix contains all of the information about the dynamics.

For each Markov process, the set of possible states can be placed in one-to-one correspondance with a subset of the non-negative integers $\{1,\dots ,d\}$. The off-diagonal elements of the rate matrix, ${\mathsf {Q}}$, are all non-negative, and the *i*th diagonal element is ${\mathsf {Q}}_{ii}=-\sum _{j=1,j\ne i}^d{\mathsf {Q}}_{i,j}$. A chain that is currently in state *i* leaves this state upon the first event of a Poisson process with a rate of $-{\mathsf {Q}}_{i,i}$; the state to which it transitions is *j* with a probability of ${\mathsf {Q}}_{i,j}/(-{\mathsf {Q}}_{i,i})$. Whilst the rate matrix, ${\mathsf {Q}}$, is a natural description of the process, the likelihood for typical observation regimes involves the transition matrix, $\exp ({\mathsf {Q}}t)$, the (*i*, *j*)th element of which is exactly ${\mathbb {P}} \left( {X_t=j|X_0=i}\right)$.

Both examples take the form of a *reaction network*, where from the current state $X_t$, the next state change will occur according to one of the specified reactions. The state can be thought of as an integer vector, where each element of the vector indicates the numbers of a particular species that are currently present in the system. When, as here, the maximum number of each species is finite, the set of possible states can be placed in one-to-one correspondence with the natural numbers as required to define ${\mathsf {Q}}$. Each reaction occurs according to a Poisson process with a rate, $\lambda (X_t)$, and when it occurs species combine according to the reaction formula. For example, the first reaction in the SIR model, below occurs with a rate of $\beta SI$, and when it occurs the state (*S*, *I*) changes to $(S-1,I+1)$.

#### Example 1

**The SIR model for epidemics**. The SIR model for a disease epidemic has 3 species: those who are susceptible to the epidemic, ${\mathsf {S}}$, those both infected and infectious, ${\mathsf {I}}$, and those who have recovered from the epidemic and play no further part in the dynamics, ${\mathsf {R}}$. The non-negative counts of each species are denoted by *S*, *I*, and *R*. For relatively short epidemics the population, $n_{pop}$, is assumed to be fixed, and so the state of the Markov chain, represented by (*S*, *I*), is subject to the additional constraint of $S+I\le n_{pop}$, with $R=n_{pop}-S-I$. The two possible reactions and their associated rates are:

$$\begin{aligned} {\mathsf {S}}+{\mathsf {I}}{\mathop {\longrightarrow }\limits ^{\beta SI}}2{\mathsf {I}}, ~~~\text{ and }~~~ {\mathsf {I}}{\mathop {\longrightarrow }\limits ^{\gamma I}}{\mathsf {R}}. \end{aligned}$$

#### Example 2

**The Moran model for allele frequency** descibes the time evolution of the frequency of two alleles, $A_1$ and $A_2$ in a population with a fixed size of $n_{pop}$. Individuals with allele $A_1$ reproduce at a rate of $\alpha$, and those with $A_2$ reproduce at a rate of $\beta$. When an individual dies it is replaced by the offspring of a parent chosen uniformly at random from the whole population (including the individual that dies). The allele that the parent passes to the offspring usually matches its own, however as it is passed down an allele may mutate; allele $A_1$ switching to $A_2$ with a probability of *u* and $A_2$ switching to $A_1$ with a probability of *v*. Let ${\mathsf {A}}_1$ and ${\mathsf {A}}_2$ represent individuals with alleles $A_1$ and $A_2$ respectively and let *N* be the number of individuals with allele $A_1$. The two reactions are

$$\begin{aligned} {\mathsf {A}}_1{\mathop {\longrightarrow }\limits ^{\lambda _N}}{\mathsf {A}}_2 ~~~\text{ and }~~~ {\mathsf {A}}_2{\mathop {\longrightarrow }\limits ^{\mu _N}}{\mathsf {A}}_1. \end{aligned}$$

Setting $f_N=N/n_{pop}$, the corresponding infinitesimal rates are

$$\begin{aligned} \lambda _N= & {} \left( 1-f_N\right) \left[ \alpha f_N(1-u)+\beta (1-f_N)v\right] ~~~\text{ and }~~~ \\ \mu _N= & {} f_N\left[ \beta (1-f_N)(1-v)+\alpha f_N u\right] , \end{aligned}$$

where the unit of time is the expectation of the exponentially distributed time for an individual to die and be replaced. $\square$

The many other examples of interest include the SIS and SEIR models for epidemics (e.g. Andersson and Britton 2000), dimerisation and the Michaelis-Menten reaction kinetics (e.g. Wilkinson 2012). Further examples but with an infinite statespace include the Schlögel model (e.g. Vellela and Qian 2009), the Lotka-Volterra predator-prey model (e.g. Wilkinson 2012, Drovandi and McCutchan 2016) and models for the autoregulation of the production of a protein (e.g. Wilkinson 2012), all of which are tackled using matrix exponentials in Sherlock and Golightly (2019).

## Data and likelihood calculations

Denote the statespace of the Markov chain $\{X_t\}_{t\ge 0}$ by ${\mathcal {X}}=\{x^{(k)}\}_{k=1}^{d}$. Let the prior mass function across states be $\nu (x|\theta )$ and define $\nu (\theta ):=(\nu (x^{(1)}|\theta ),\dots ,\nu (x^{(d)}|\theta ))$. Let the infinitesimal generator be ${\mathsf {Q}}(\theta )$, and suppose there are observations $y_0,y_1,\dots ,y_n$ at times $t_0,t_1,\dots ,t_n$, where $Y_i|(X_i=x_i)$ has a mass function of $p(y_i|x_i,\theta )$, $i=0,\dots ,n$.

### Likelihood for noisy and partially observed data

For any continuous-time Markov chain $\{X_t\}_{t\ge 0}$ with an infinitesimal generator, or rate matrix of ${\mathsf {Q}}$, the $(x,x')th$ element of $\exp ({\mathsf {Q}}t)$ gives the transition probability (e.g. Norris (1997)):

$$\begin{aligned} {\mathbb {P}} \left( {X_t=x'\mid X_0=x}\right) =\left[ \exp ({\mathsf {Q}}t)\right] _{x,x'}, \end{aligned}$$

where here and elsewhere we abuse notation by identifying the state $x^{(i)}\in {\mathcal {X}}$ with the corresponding index $i\in \{1,\dots ,d\}$.

Defining the diagonal likelihood matrix to be $L_j(\theta )=\mathsf {diag}(p(y_j|x^{(1)},\theta ),\dots ,p(y_j|x^{(d)},\theta ))$ and $\Delta _j=t_j-t_{j-1}$, $j=1,\dots ,n$, the likelihood for the observations is then

1 $$\begin{aligned} {\mathbb {P}} \left( {y_0,\dots ,y_n\mid \theta }\right)&= \sum _{(x_0,\dots ,x_n)\in {\mathcal {X}}^{n+1}} {\mathbb {P}} \left( {X_0=x_0}\right) {\mathbb {P}} \left( {Y_0=y_0|X_{0}=x_0}\right) \nonumber \\&\prod _{j=1}^n{\mathbb {P}} \left( {X_j=x_j| X_{j-1}=x_{j-1}}\right) {\mathbb {P}} \left( {Y_j=y_j|X_{j}=x_j}\right) \nonumber \\ =&\, \nu (\theta )^\top L_0(\theta )\left[ \prod _{j=1}^n \exp ({\mathsf {Q}}(\theta ) \Delta _j)L_j(\theta )\right] {\underline{1}}, \end{aligned}$$

where ${\underline{1}}$ is the *d*-vector of ones. Similarly, the filtering distribution after observation $y_m$ is

2 $$\begin{aligned} {\mathbb {P}} \left( {X_{t_m}=x\mid y_0,\dots ,y_m}\right)&= \frac{ \nu (\theta )^\top L_0(\theta )\left[ \prod _{j=1}^m \exp ({\mathsf {Q}}(\theta ) \Delta _j)L_j(\theta )\right] }{ \nu (\theta )^\top L_0(\theta )\left[ \prod _{j=1}^m \exp ({\mathsf {Q}}(\theta ) \Delta _j)L_j(\theta )\right] {\underline{1}}}. \end{aligned}$$

Consider the required multiplication from left to right: since the likelihood vectors $L_j(\theta )$ are diagonal, pre-multiplication by a *d*-vector is an ${\mathcal {O}}(d)$ operation. Pre-multiplication of the exponential of a sparse matrix by a *d*-vector via the uniformisation algorithm is also ${\mathcal {O}}(d)$ (see Sect. 3.1), so the entire likelihood calculation is ${\mathcal {O}}(d)$. In the case of certain epidemic models *d* itself can be much smaller than might naively be assumed.

### Statespace reduction for epidemic models

An alternative formulation of the statespace of the Markov chain for an SIR epidemic model (or more general models such as the SEIR), in terms of the *degree of advancement* (DA), was first pointed out in Jenkinson and Goutsias (2012). Instead of representing the state in terms of the number of susceptibles and the number of infecteds, given a known initial condition it is represented by the number of new infections and the number of new removals, $B_I$ and $B_R$, neither of which can be negative and both of which are non-decreasing. Given the initial condition, the map from (*S*, *I*) to $(B_I,B_R)$ is one-to-one; however the rate matrix with the DA formulation is lower triangular, a key ingredient in the implicit Euler integration scheme used in Jenkinson and Goutsias (2012) to integrate the master equation.

When performing Bayesian inference for the SIR model using noisy, partial observations, Ho et al. (2018) points out that augmenting the state space of the MCMC Markov chain to include not just the model parameters but also the true values of *S* and *I* at each observation time can massively reduce the sizes of the statespaces that need to be considered when evolving the SIR process from one observation time to the next provided the DA formulation is used. Consider the case of exact observations and suppose, for example, that in a population of size $n_{pop}=500$, $x_a=(S_a,I_a,R_a)=(485,2,13)$ and for some $t>0$, $x_{a+t}=(470,3,27)$. Then $b_R=R_{a+t}-R_a=14$ and $b_I=S_{a}-S_{a+t}=15$. The size of the statespace for evolution between time *a* and time $a+t$, ${\mathcal {X}}_{a}^{a+t}$, is then reduced from the size of the full statespace, $(n_{pop}+1)(n_{pop}+2)/2=125751$ to $(b_I+1)(b_R+1)=240$. The exponential of the rate matrix is not used in Ho et al. (2018); instead, a recursive formula for the Laplace transform of the transition probability to a given new state in terms of transition probabilities for old states then permits estimation of the transition vector from a known initial starting point in ${\mathcal {O}}(d)$ operations, where *d* is the dimension of the statespace actually required. Inference is then performed for the SIR model using data from the Ebola outbreak in regions of Guinea.

We may use the DA formulation with data augmentation, provided we include an additional coffin state, ${\mathsf {C}}$, with $Q_{{\mathsf {C}},x}=0$ for all $x\in {\mathcal {X}}_a^{a+t}\cup {\mathsf {C}}$. Any births that would leave the statespace (and hence contradict the observation at time $a+t$) instead go to ${\mathsf {C}}$. The aforementioned implementation, a square grid of possible states, includes “impossible” states to which the rate of entry is zero: the current number of infections can never be negative, so, throughout the time interval $[a,a+t]$, $b_R\le I_a+b_I$. Removing these states altogether allows us to make a further reduction in the size of the statespace, by a factor of up to one half. In the example above, this reduces the statespace size still further, from 240 to 162.

## Matrix exponentiation

The exponential of a $d\times d$ square matrix, ${\mathsf {M}}$ is defined via its infinite series: $e^{\mathsf {M}}=\sum _{i=0}^\infty \frac{1}{i!}{\mathsf {M}}^i$. As might be anticipated from the definition, for a $d\times d$ matrix, algorithms for evaluating $\exp {({\mathsf {M}})}$ take ${\mathcal {O}}(d^3)$ operations (see Moler and Van Loan (2003), for a review of many such methods). However, for a *d*-vector, *v*, the product $\exp {(Mt)}~v$ is the solution to the initial value problem $w(0)=v$, $dw/dt={\mathsf {M}}w$, and is the key component of the solution to more complex differential equations such as $dw/dt={\mathsf {M}}w +B u(t)$. For this reason the numerical evaluation of the action of a matrix exponential on a vector has received considerable attention of itself, e.g. Gallopoulos and Saad (1992), Saad (1992), Sidje (1998), Al-Mohy and Higham (2011).

When ${\mathsf {M}}$ is dense,

3 $$\begin{aligned} \exp ({\mathsf {M}})~v=\sum _{i=0}^\infty \frac{1}{i!}{\mathsf {M}}^iv \end{aligned}$$

can be evaluated in ${\mathcal {O}}(d^2)$ operations if the series is truncated at an appropriate point. However, motivated by the examples in Sect. 1.1 our interest lies in large *sparse* matrices, and the number of operations can then be reduced to ${\mathcal {O}}(rd)$, where *r* is the average number of entries in each row of ${\mathsf {M}}$.

With double-precision arithmetic, real numbers are stored to an accuracy of approximately $10^{-16}$. Thus, evaluation of the exponential of a large negative number via its Taylor series is prone to potentially enormous round-off errors due to the almost cancellation of successive large positive and negative terms; a similar problem can affect the exponentiation of a matrix. Such issues are typically circumvented via the identity

4 $$\begin{aligned} \exp ({\mathsf {M}})v&= \left[ \prod _{k=1}^K \exp ({\mathsf {M}}/K)\right] v, \end{aligned}$$

applied for a sufficiently large integer *K*, and evaluated via *K* successive evaluations of product of $\exp ({\mathsf {M}}/k)$ and a vector. The calculation on the right of (4) typically involves many more numerical operations than the direct calculation on the right of (3), so *K* should be the *smallest* integer that leads to the required precision by mitigating sufficiently against the cancellation of large positive and negative terms. This minimises both the accumulation of rounding errors and the total compute time given the required accuracy.

One common technique for such multiplication, exemplified in the popular Expokit FORTRAN routines of Sidje (1998), estimates $e^{{\mathsf {M}}/K}v$ via its projection on to the Krylov subspace of $\mathsf {Span}\{v, {\mathsf {M}}v,\dots ,{\mathsf {M}}^{n-1}v\}$, where $n<<d$. A second method is provided in Al-Mohy and Higham (2011), where the key contributions lie in the method for choosing *K* and for choosing a suitable truncation point for the infinite series, as well as a means of truncating each series early depending on the behaviour of recent terms. These and other algorithms are compared, specifically for the case of the SIR model (which has a special structure; see Sect. 2.2) in Kinyanjui et al. (2018).

Both Krylov methods and that of Al-Mohy and Higham (2011) use the fact that ${\mathsf {M}}$ is sparse and that only the action of $\exp ({\mathsf {M}})$ on a vector is required, but neither uses the structure of interest to us: we require $\nu ^\top \exp ({\mathsf {Q}}t)$ where ${\mathsf {Q}}$ is a rate matrix for a general MJP and $\nu$ is a non-negative vector. Since ${\mathsf {Q}}t$ is also a rate matrix, we henceforth set $t=1$ without loss of generality. Let

5 $$\begin{aligned} \rho :=\max _{i=1,\dots ,d} |{\mathsf {Q}}_{ii}| ~~~\text{ and }~~~ {\mathsf {P}}=(1/\rho ){\mathsf {Q}}+I. \end{aligned}$$

${\mathsf {P}}$ is a Markov transition matrix, and the key observation is that

6 $$\begin{aligned} \exp {\mathsf {Q}}=\exp (\rho {\mathsf {P}}-\rho {\mathsf {I}}) =\exp (-\rho )\exp (\rho {\mathsf {P}}) =\sum _{i=0}^\infty \exp (-\rho )\frac{\rho ^i}{i!}{\mathsf {P}}^i. \end{aligned}$$

Firstly, ${\mathsf {P}}$ has no negative entries so cancellation of terms with alternating signs is no longer a concern. Secondly, $\exp {\mathsf {Q}}$ can be interpreted as a mixture over a $\text{ Poisson }(\rho )$ random variable *I*, of *I* transitions of the discrete-time Markov chain with a transition matrix of ${\mathsf {P}}$.

The next two subsections detail variations on two existing algorithms that utilise this special structure: the *uniformisation* algorithm and a variation on the *scaling and squaring* algorithm. For sparse rate matrices, the uniformisation algorithm has a cost of ${\mathcal {O}}(\rho d)$, whereas the scaling and squaring algorithm has a cost of ${\mathcal {O}}(d^3\log \rho )$. Thus, the uniformisation algorithm is preferred when $\rho$ is small, and scaling and squaring when $\rho$ is large but *d* is relatively small. We now describe the two algorithms in detail.

### The uniformisation algorithm

In many statistical applications, the most appropriate algorithm for calculating $\mu ^\top :=\nu ^\top \exp {\mathsf {Q}}$ is the uniformisation algorithm, e.g. Reibman and Trivedi (1988), Sidje and Stewart (1999). This estimates $\mu ^\top$ by truncating a single series none of whose terms can be negative, rather than truncating multiple series where terms may change sign as in Al-Mohy and Higham (2011). Given an $\epsilon >0$, the algorithm calculates an approximation, ${\widehat{\mu }}$, to $\mu$ by picking a truncation point for the infinite series, such that, if $\nu$ were a probability vector, the (guaranteed to be non-negative) amount of *true* missing probability over all of the *d* dimensions is controlled:

$$\begin{aligned} 0< 1-\frac{||{\widehat{\mu }}^*||_1}{||\nu ||_1} < \epsilon , \end{aligned}$$

where ${\widehat{\mu }}^*$ is the probability vector that would be calculated if there were no rounding errors, and the only errors were due to the truncation of the infinite series. Typically we aim for $\epsilon$ to be similar to the machine’s precision. We control the absolute truncation error and note that with any truncation of the power series, it is impossible to obtain general control of the *relative error in a given component* of $\mu$, $|{\widehat{\mu }}_i/\mu _i-1|$. Consider, for example, a Moran process (Example 2), where ${\mathsf {Q}}$ is tridiagonal. Then ${\mathsf {Q}}^k$ is also banded, with a band width of $2k+1$. For any given $m_{max}$, and $\nu =(1,0,0,\dots )$, set $d>m_{max}+1$. The truncated approximation to $e^{{\mathsf {Q}}}$ gives a transition probability of 0 for all states above $m_{max}+1$, yet, in truth there is a non-zero probability of such a transition. However, the combined probability of all transitions which have not been accounted for is, by design, at most $\epsilon$.

From (6),

$$\begin{aligned} \mu ^\top =\nu ^\top e^Q = e^{-\rho }\nu ^\top \sum _{i=0}^\infty \frac{\rho ^i}{i!}P^i \approx e^{-\rho } \sum _{i=0}^{m} \frac{\rho ^i}{i!}\nu ^\top P^i=:{\widehat{\mu }}^{*\top }. \end{aligned}$$

Now,

$$\begin{aligned} \sum _{i=1}^d {\widehat{\mu }}^*_i = {\widehat{\mu }}^{*\top }1 = e^{-\rho }\sum _{i=0}^m \frac{\rho ^i}{i!}\nu ^\top P^i 1 = ||\nu ||_1 e^{-\rho }\sum _{i=0}^m \frac{\rho ^i}{i!}. \end{aligned}$$

So the absolute relative error, or (when $\nu$ is a probability vector) missing probability mass, due to truncation is

$$\begin{aligned} r_m(\rho ):=e^{-\rho }\sum _{i=m+1}^\infty \frac{\rho ^i}{i!}, \end{aligned}$$

the tail probability of a $\mathsf {Poisson}(\rho )$ random variable. Of direct interest to us is

$$\begin{aligned}m_{\epsilon }(\rho ) := \inf \{m\in {\mathbb {N}}: r_m(\rho )\le \epsilon \}, \end{aligned}$$

the smallest *m* required to achieve an error of at most $\epsilon$, or, essentially, the quantile function for a $\mathsf {Poisson}(\rho )$ random variable, evaluated at $1-\epsilon$. Chebyshev’s inequality applied to $X/\rho$, where $X\sim \mathsf {Poisson}(\rho )$ gives ${\mathbb {P}} \left( {|X/\rho - 1|\ge 1/\sqrt{\epsilon \rho }}\right) \le \epsilon$, implying the $m={\mathcal {O}}(\rho )$ computational cost given earlier in this section.

In many programming languages, standard functions are available to evaluate $m_{\epsilon }(\rho )$. However, for example, in R we find
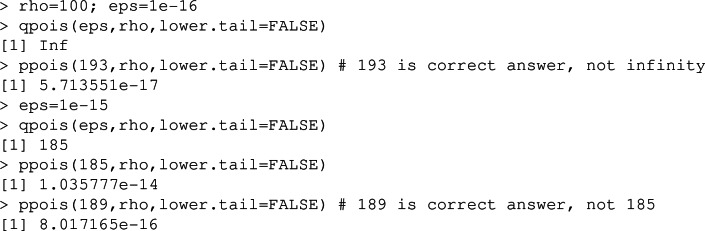
*i.e.*, an inability to calculate $m_{\epsilon }(\rho )$ correctly given the small $\epsilon$ values that we require; the underlying functions are also callable from C++ and lead to the same error. In Appendix A we provide sharp bounds on $m_{\epsilon }(\rho )$, and this leads to an accurate methodology for its exact calculation, producing the same (correct) answers as the C++ boost library (which we have not been able to use with Rcpp) and up to twice as quickly.

The uniformisation algorithm is presented as Algorithm 3.1. For large values of $\rho$, although there is no problem with large positive and negative terms cancelling, it is possible that the partial sum $\sum _{i=0}^k \frac{\rho ^i}{i!}$ might exceed the largest floating point number storable on the machine. We circumvent this problem by occasionally renormalising the vector partial sum when the most recent contribution is large, and compensating for this at the end; see lines 5, 12 and 14.
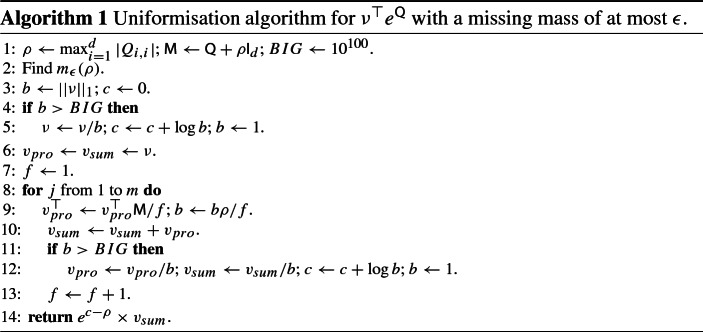


### Scaling and squaring

One of the simplest, yet most robust methods for exponentiating any square matrix is the scaling and squaring algorithm, e.g. Moler and Van Loan (2003). When the square matrix is an infinitesimal generator, this method can be made even more robust using the reformulation in (6). Furthermore, when not $\exp {\mathsf {Q}}$ but $\nu ^\top \exp {\mathsf {Q}}$ is required, some further computational savings can be obtained.

The basic scaling and squaring algorithm takes advantage of the identity

$$\begin{aligned} \exp ({\mathsf {M}})=\left[ \exp ({\mathsf {M}}/2^s)\right] ^{2^s}, \end{aligned}$$

where for any integer *s*, a square matrix is raised to the power of $2^s$ by squaring it *s* times. We set ${\mathsf {M}}={\mathsf {Q}}+\rho {\mathsf {I}}=\rho {\mathsf {P}}$ from (5). And define ${\mathsf {M}}_{small}={\mathsf {M}}/2^s$. First, $\exp ({\mathsf {M}}_{small})$ is approximated via the uniformisation algorithm applied to a matrix, e.g. Ross (1996): $\sum _{i=0}^m {\mathsf {M}}_{small}^i/i!$. This quantity is then squared *s* times. The optimal value of *s* is chosen according to an algorithm described in Appendix B.

When evaluating $\nu ^\top \exp ({\mathsf {Q}})=\exp (-\rho )\nu ^\top \exp ({\mathsf {M}})$ via scaling and squaring with $s>0$ it is never most efficient to first evaluate $\exp ({\mathsf {M}})$. Let $s_1$ and $s_2$ be two integers such that $s_1+s_2=s$. Then

$$\begin{aligned} \nu ^\top \exp ({\mathsf {M}}) = \nu ^\top [\exp ({\mathsf {M}}_{small})]^{2^{s_1}}[\exp ({\mathsf {M}}_{small})]^{2^{s_1}}\dots [\exp ({\mathsf {M}}_{small})]^{2^{s_1}}, \end{aligned}$$

with $2^{s_2}$ matrix vector products. The cost of $s_1$ matrix squares and $2^{s_2}$ vector-matrix products (where the matrix is dense) is $s_1d^3+2^{s_2}d^2$. We round the minimiser down to the nearest integer for simplicity, setting

7 $$\begin{aligned} s_2=\min \left( s,\lfloor (\log d-\log \log 2) /\log 2\rfloor \right) \end{aligned}$$

Even with $d=2$ this gives $s_2=\min (s,1)$.
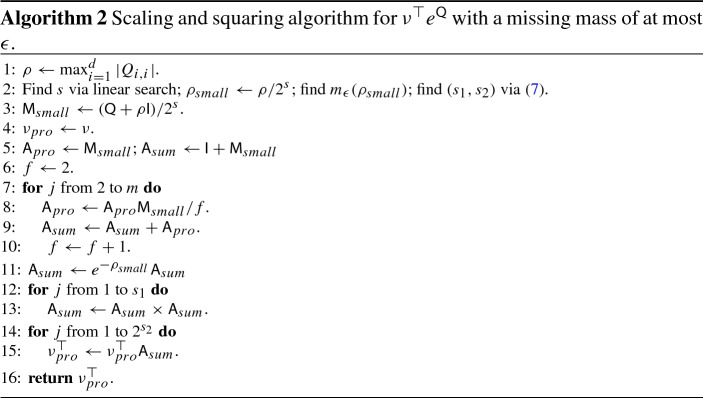


### Improvements

We now describe two optional extensions: renormalisation, which improves the accuracy of any matrix exponentiation algorithm used on a rate matrix, and two-tailed truncation, which is unique to the uniformisation algorithm and allows a small computational saving.

Since $a:=\sum _{i=1}^d \mu _{i}=\sum _{i=1}^d \nu _{i}$ there is no need to keep track of the logarithmic offset (*c* in Algorithm 3.1). Instead the final vector ($v_{sum}$ in Algorithm 3.1) is renormalised at the end so that its components sum to *a*.

Two-tailed truncation, e.g. Reibman and Trivedi (1988) permits a small reduction in the computational cost of the uniformisation algorithm with no loss of accuracy. When $\rho$ is moderate or large, the total mass of probability from the initial value of $v_{sum}$ and the early values accumulated into $v_{sum}$ (Steps 6 and 10 of Algorithm 3.1) is negligible (has a relative value smaller than $\epsilon /2$, say) compared with the sum of the later values. In such cases $v_{sum}$ may be initialised to 0 and step 10 omitted for values of *j* beneath some $m_{lo}$. Proposition 1 below shows that if *m* is chosen such that ${\mathbb {P}} \left( {{{\mathsf {P}}}{{\mathsf {o}}}(\rho )>m}\right) \le \epsilon /2$ then setting $m_{lo}:=\max (0,2\lfloor \rho -0.5\rfloor -m)$ ensures that the missing probability mass is no more than $\epsilon$. For large $\rho$, $m-m_{lo}={\mathcal {O}}(\sqrt{\rho })$, so with two-tailed truncation the cumulative cost of Step 10 dwindles compared with the other ${\mathcal {O}}(d)$ costs, which are repeated ${\mathcal {O}}(\rho )$ times.

#### Proposition 1

Given $\rho >0$, let $p_n=e^{-\rho }\rho ^n/n!={\mathbb {P}} \left( {\mathsf {Poisson}(\rho )}\right) =n$, and let $c=\lfloor \rho -1/2\rfloor$. Then for $a\le c-1$,

$$\begin{aligned} \sum _{j=0}^{c-a-1}p_j < \sum _{j=c+a+1}^\infty p_j. \end{aligned}$$

#### Proof

For any integer *b*, and $1\le i \le b$,

$$\begin{aligned}&\frac{p_{b-i}}{p_{b+i}}\\&\quad =\rho ^{-2i}b(b+1)(b-1)(b+2)\dots (b-i+1)(b+i)\\&\quad = \rho ^{-2i}\left[ b_*^2-\frac{1}{2^2}\right] \cdots \left[ b_*^2-\frac{(2i-1)^2}{2^2}\right] \end{aligned}$$

where $b_*=b+1/2$. Hence, if $b_*\le \rho$, $p_{b-1}/p_{b+i}<1$, and so

$$\begin{aligned} \sum _{j=0}^{\lfloor b^*\rfloor -a-1} p_i= \sum _{i=a+1}^{\lfloor b_*\rfloor } p_{b-i}< \sum _{i=a+1}^{\lfloor b_*\rfloor } p_{b+i}<\sum _{i=a+1}^{\infty }p_{b+i}. \end{aligned}$$

$$\square $$

### Implementation

Our C++ implementation uses the recent basic sparse matrix functionality in the C++ Armadillo library; see Sanderson and Curtin (2016), Sanderson and Curtin (2018) to calculate $\nu ^\top \exp {\mathsf {Q}}$, where $\nu$ is non-negative and ${\mathsf {Q}}$ is a large, sparse rate matrix. Direct function calls from the R programming language are enabled through RcppArmadillo; see Eddelbuettel and Sanderson (2014). For completeness, the functions can also be called with dense rate matrices. The functions are collected into the expQ package which is downloadable from https://github.com/ChrisGSherlock/expQ and are briefly outlined in Appendix C.

The speed of a vector multiplication by a sparse-matrix depends on the implementation of the sparse matrix algorithm. In RR Core Team (2018) and in C++ Armadillo, sparse matrices are stored in column-major order. Hence pre-multiplication of the sparse matrix by a vector, $\nu ^\top Q$, is much quicker than post multiplication, $Q\nu$. In other languages, such as Matlab, sparse matrices are stored in row-major order and post-multiplication is the quicker operation, so $Q^\top$ should be stored and used, rather than *Q*.

## Numerical comparisons and demonstrations

In Al-Mohy and Higham (2011) their new algorithm (henceforth referred to as AMH) is compared across many examples against state-of-the-art competitors, including, in particular, the expokit function expv of Sidje (1998). In most of the experiments AMH is found to give comparable or superior accuracy together with superior computational speed. Given these existing comparisons and that the superiority of the uniformisation algorithm over the algorithm of Al-Mohy and Higham (2011) (for rate matrices) is not the main thrust of this paper, we perform a short comparison of accuracy and speed for two different likelihood calculations for an SIR model fitted to data from the Eyam plague. We compare our implementation of the uniformisation algorithm, the algorithm of AMH, the expAtv function which is from the R package expm and uses the method of Sidje (1998), and the bespoke algorithm for epidemic processes in Ho et al. (2018). Since it would be unfair to compare the clock-speeds for the Matlab code for AMH directly with those of our RcppArmadillo implementation, we compare the number of sparse vector-matrix multiplications that are required.

When performing maximum-likelihood estimation, each iteration of the optimisation algorithm tries a new parameter value, and when performing Bayesian inference, each iteration of the algorithm proposes a new parameter value. In each case, given the parameter value, the pertinent rate matrices are created and then the matrix exponentiation function is called in turn for each of the matrices as required by (1). If the generic exponentiation function is called then the decision on whether to use Algorithm 1 or Algorithm 2 is based upon the dimension, *d* and the maximum absolute value on the diagonal, $\rho$. Whether Algorithm 1 or 2 is called directly or via the generic exponentiation function, the first task it performs is the evaluation of $\rho$. The cost of this is neglibible compared with that of the exponentiation itself, so it is essentially immaterial that $\rho$ is evaluated twice when the generic exponentiation function is called.

The highest accuracy available in C++ using sparse matrices and the armadillo linear algebra library is double precision, which we used throughout in our implementation of both of our algorithms. For the uniformisation and scaling and squaring algorithms we used $\epsilon =10^{-15}$, and for AMH we used the double-precision option. For expAtv and for Ho et al. (2018) we use the default package setting.

### Comparison with other matrix exponentiation algorithms

To examine the speed and accuracy of the algorithm we consider the collection (see the first three rows of Table 1) of (*S*, *I*) (susceptible and infected) values, which originated in Raggett (1982) and were used in Ho et al. (2018), for the Eyam plague. We set the parameters to their maximum-likelihood estimates, $(\beta ,\gamma )=(0.0196,3.204)$ and consider the likelihood for the data in Table 1. In addition, to mimic the size of potential changes between observation times and the size of the elements of the rate matrix from a larger population, we also evaluated the likelihood for the jump directly from the data at time 0 to the data at time 4. The final three rows of Table 1 refer to the rate matrix for the transition between consecutive observations and provide the dimension the matrix first using the reformulation of Ho et al. (2018) and then applying the improvement described in Sect. 2.2; the final row is the absolute value of the largest entry of ${\mathsf {Q}}$, $\rho$. The rate matrix for the single jump between times 0 and 4 had $d_{HCS}=30789$, $d=16082$ and $\rho \approx 3439.5$. The full statespace has a size of 34453. Thus, for large changes, the main reduction in size arises from the improvement in Section 2.2, but for small jumps this provides a smaller relative reduction compared with that in Ho et al. (2018).

**Table 1:** Time (in units of 31 days), and numbers of susceptibles and infecteds, originally from Raggett (1982). The final rows indicates, for each pair of consecutive observations, the size of the statespace for evaluating the transition probability and the $\rho$ value for the associated rate matrix

Time	0	0.5	1.0	1.5	2.0	2.5	3.0	4.0
S	254	235	201	153	121	110	97	83
I	7	14	22	29	20	8	8	0
$d_{HCS}$	-	261	946	2059	1387	289	197	346
*d*	-	245	867	1868	1308	282	181	240
$\rho$	-	101.5	171.4	217.1	170.1	83.1	53.6	106.3

For the uniformisation and scaling and squaring algorithm, with $\epsilon =10^{-15}$, the algorithm of Ho et al. (2018) and the expAtv function from the R package expm which uses the technique of Sidje (1998) we found the CPU time for 1000 estimations of the likelihood (20 estimates for the likelihood for the jump from $t=0$ to $t=4$). We also recorded the error in the evaluation of the log likelihood. Since for uniformisation, using renormalisation and two-tailed truncation together produced the fastest and most accurate evaluations, we only considered this combination. Given that the true likelihood is not known, the error using uniformisation, from scaling and squaring and from Al-Mohy and Higham (2011) were approximately bounded by examining their discrepancy from each other. The results are presented in Table 2.

**Table 2:** Timings for estimating the full log-likelihood (1000 repeats) and the log-likelihood for the jump from the initial to the final observation (20 repeats) for the Eyam data set, number of sparse vector-matrix multiplications for one repeat, and the accuracies of the estimates. Results are given for the method of Ho et al. (2018) (HCS), the expAtv function in the expm package, which uses the Krylov subspace techniques of Sidje (1998), the method of Al-Mohy and Higham (2011) (AMH), the uniformisation algorithm (Unif) and the scaling and squaring algorithm (SS). $^1$ The timing for SS on the jump likelihood was estimated from a single repeat

Algorithm	Full likelihood			Jump likelihood		
	Time (secs)	Mult	Accuracy	Time (secs)	Mult	Accuracy
HCS	45.3	–	$5.7\times 10^{-8}$	9.7	–	$4.3\times 10^{-9}$
expAtv	558.5	–	$1.6\times 10^{-10}$	323.2	–	$8.2\times 10^{-11}$
AMH	–	3701	$<1\times 10^{-15}$	-	14300	$<4\times 10^{-14}$
Unif	18.72	1596	$<1\times 10^{-15}$	15.2	3921	$<6\times 10^{-14}$
SS	1678	–	$1.1\times 10^{-13}$	8940$^1$	-	$<6\times 10^{-14}$

Scaling and squaring is extremely slow in these high-dimensional scenarios; however, Sherlock and Golightly (2019) provides a bistable example, the Schlögel model, where $d\approx 100-200$ but $\rho >10^5$, and the scaling and squaring algorithm outperforms uniformisation by orders of magnitude.

Since $m={\mathcal {O}}(\rho )$ the choice of tolerance, $\epsilon$, typically has only a small effect on the speed of the uniformisation algorithm. For the full likelihood evaluation, uniformisation is over twice as fast as the algorithm of Ho et al. (2018) and approximately thirty times as fast as expAtv, and is more accurate than either; it is also over twice as fast as the algorithm of Al-Mohy and Higham (2011), although both are very accurate.

For the single large jump between observations, we see the same pattern in terms of accuracy. There is a gain in efficiency by using two-tailed-truncation because $\rho$ is larger ($m_{lo}=3081$ and $m=3797$), but despite this, the method of Ho et al. (2018) is now more efficient than uniformisation, although considerably less accurate than it. Again, expAtv is over twenty times slower than uniformisation and less accurate, and AMH is over three times slower than uniformisation.

### Maximum likelihood inference, filtering and prediction

We now consider the Moran model, which has four unknown parameters: $(\alpha ,\beta ,u,v)$ and $n_{pop}=1000$. Setting $(\alpha ,\beta ,u,v)=(1,0.3,0.2,0.1)$, we simulate a path of the process for $T=10000$ time units. We then sample 51 observations at times $0,200,400,\dots ,10000$, by taking the value of the process at each of these times and adding independent noise with a distribution of $\mathsf {Bin}(800,0.5)-400$.

**Fig. 1:**
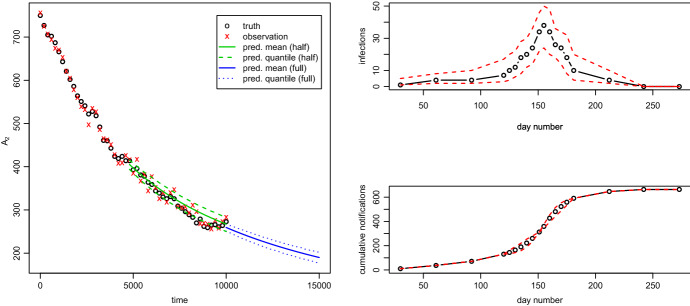
Moran model (left): true values (${\mathsf {o}}$), observations (${\mathsf {x}}$), filtering/prediction mean (solid lines) and $95\%$ quantiles (dashed and dotted lines) for a further time of 5000 from data up to $T=5000$ and data up to $T=10000$. Swansea measles SIR model (right): posterior median (solid line, with ${\mathsf {o}}$ to show the positions) and posterior $95\%$ quantiles for the number of infected people at each real or latent observation time (top) and the cumulative number recovered by that time (bottom)

We then perform inference on $\theta =(\log \alpha , \log \beta , \log [u/(1-u)], \log [v/(1-v)])$ by maximising the likelihood based on all the data and, separately, based on the data up to $T=5000$. In each of these two data scenarios we find the filtering distribution, ${\mathbb {P}} \left( {X_T|y_{0:T},{\widehat{\theta }}}\right)$, at time *T* via (2); finally we predict forward from *T* in steps of 200 for a further time of $T_{pred}=5000$ by repeatedly multiplying the current distribution vector by $\exp (200 {\mathsf {Q}}({\widehat{\theta }}))$. The true values, observations and filtering and prediction distributions are shown in Fig. 1. The whole process of inference and prediction took less than two minutes on a single i7-3770 CPU running at 3.40GHz. Further, after defining ${\mathsf {Q}}$, only 10 lines of $\texttt {R}$ code are required to calculate the log-likelihood, and fewer than this to produce the filtering distribution (see Appendix E).

### Bayesian inference for the Swansea measles epidemic of 2013

The largest measles outbreak in the United Kingdom between 2011 and 2019 centred around Swansea, Wales and occurred between November 2012 and July 2013. Of the 1219 cases in mid- and west-Wales, 664 occurred in the Swansea Local Authority (LA) area, 243 in the nearby Neath and Port Talbot LA and fewer than 80 occurred in any of the other individual LA areas in West or South Wales (http://www.wales.nhs.uk/sitesplus/888/page/66389, accessed February 10th 2020). A reduction in uptake of the MMR (Measles, Mumps, Rubella) vaccine has been blamed e.g. Jakab and Salisbury (2013) for this, with particularly low rates reported in Swansea (https://en.wikipedia.org/wiki/2013_Swansea_measles_epidemic, accessed February 10th 2020).

The basic reproduction number, $R_0$, is the expected number of secondary infections in a susceptible population that arise directly from the primary infection of a single individual. For the SIR model described in Section 1.1, $R_0=\beta /\gamma$. For measles, $R_0$ is often reported as between 14 and 18 (e.g. Anderson and May (1982)), which fits with the World Health Organisation (WHO) recommendation of vaccination level of at least $93-95\%$ WHO (2009).

We fit the SIR model to the notification data for the Swansea LA provided in Table 3 so as to estimate the overall $R_0$ for the partially vaccinated population in Swansea and to demonstrate inference on the unknown number of infectious individuals at each observation time. In fitting the model we are making several assumptions and simplifications, including the following. Firstly, we are ignoring infections from Swansea to other LAs and from these LAs to Swansea; since most of the infections occurred in Swansea the former will outnumber the latter and so we will underestimate the ‘true’ $R_0$, and provide a ‘local’ $R_0$ at the epicentre of the infection. Secondly it is known that the lowest level of vaccination, and the highest level of infection was amongst 10-18 year olds, see Wise (2013); a more accurate model would, therefore, partition the population into age groups. Age-stratified, continuous-time Markov chain SIR models are difficult to fit in general, however, and often a deterministic version of the model is used (e.g. Broadfoot and Keeling (2015)). Finally, we treat a notification as equivalent to a removal: this is not unreasonable as once an individual has been diagnosed by a GP with suspected measles they will be asked to isolate themselves.

**Table 3:** Number of measles notifications in the Swansea Local Authority area by month (from http://www.wales.nhs.uk/sitesplus/888/page/66389, February 10th 2020)

Month	2012			2013						
	Oct	Nov	Dec	Jan	Feb	Mar	Apr	May	Jun	Jul
Day number	0	30	61	92	120	151	181	212	242	273
Notifications	0	10	27	34	59	183	278	56	17	0

As described in Section 2.2 we add as latent variables the number of infections at each of the reporting times, Days 30, 61, 92, ..., 212. The number of infections at times 242 and 273 must both be zero.

To understand the evolution near the peak of the epidemic and speed up inference still further, we add latent observation times during the peak of the infection, at Days 125, 130, 135, 140, 145, 155, 160, 165, 170 and 175. This leads to 10 further latent observations of the number of infected individuals and (because of constraints) 10 further latent observations of the number removed during each reduced time period, leading to a total of 27 integer

latent variables.

We use a ${\mathsf {N}}(\log 5,2/3)$ prior for $\log R_0=\log (\beta /\gamma )$, a ${\mathsf {N}}(\log (1/15),1)$ prior for $\log \gamma$ and, because it is very poorly identified, we set the prior for the effective population size to $p(N_{pop}=n)\propto \exp (-n/500)1_{\{n\ge 1000\}}$.

We perform inference via a Metropolis-within-Gibbs algorithm: $\theta =(\log \beta , \log \gamma )$ is updated via a random walk proposal with a jump of ${\mathsf {N}}(0,\lambda ^2 {\mathsf {I}}_2)$, $n_{pop}$ via an integer-valued random walk proposal, and $x_{latent}$, the latent observations via integer random walks, with physical constraints (such as the sum of all the *R*s not being able to exceed $n_{pop}$) checked for automatically; see Appendix D for more details.

The basic reproduction number, $R_0$, is estimated as 1.15, with a $95\%$ credible interval of (1.01, 1.31). This fits with other information known: firstly,up until 2013, $R_0$ only changed gradually over time (due to year-on-year variations in infant vaccination rates) and it cannot have reached much higher than 1 in late 2012 as otherwise there would have been an outbreak in a previous year; secondly an $R_0$ of 1.15 if the true $R_0$ is 16, corresponds to a vaccination level of $93\%$, and $R_0=1.3$ corresponds to a $92\%$ level, and as argued earlier, we expect to slightly underestimate $R_0$. As of December 2012, the estimated coverage of one dose of MMR vaccine among 16 year-olds in Wales was $91\%$; see Public Health Wales (2013).

The right-hand panels of Fig. 1 show the posterior median and $95\%$ credible intervals for the number of infections at each of the monthly observation times and at the 10 additional latent times, and similar intervals for the cumulative number of infections. In any infectious disease, at any current time point, it is vital to understand the current, unknown, number of infections in order to be able to predict the future course of an epidemic.

## Discussion

We have shown that inference, prediction and filtering for continuous-time Markov chains with a large but finite statespace, especially those arising from reaction networks is not just conceptually straightforward when the matrix exponential is used, but it is also often practical. We have provided and demonstrated the use of robust tools for this purpose in R, which opens up the direct use of and inference for reaction-network models to a wider audience. Straightforward inference for epidemic models, such as the SIR and SEIR models is particularly apposite at the time of submission, as it might have enabled an analysis of early COVID-19 infection data by people not expert in the more complex MCMC methodology typically used.

We emphasise that we are not suggesting that the tools we provide should replace the particle MCMC, ABC and SMC methods currently employed. In our experience, inference for epidemic models coded in a fast, compiled language is often more efficient in terms of effective samples per second, for example, than the approach using matrix exponentiation. However, the matrix exponential approach *is* much more straightforward, and the code that uses it can be written in the simpler, interpreted language R.

As the size of the statespace increases, the efficiency of the matrix exponentiation approach decreases; however, once the statespace becomes sufficiently large, the evolution of the process is often approximated by a stochastic differential equation (e.g. Golightly and Wilkinson 2005, Fearnhead et al. 2014) or, when the behaviour is effectively deterministic, by ordinary differential equations (e.g. Broadfoot and Keeling 2015).

For the scaling and squaring approach, in particular, the cost of the exponentiation of ${\mathsf {Q}}/K$ can be nearly halved by using a Padé approximant (e.g. Moler and Van Loan (2003)), but this then requires a matrix inversion, and so, for reasons of robustness, was not pursued here.

## Figures and Tables

**Fig. 2:**
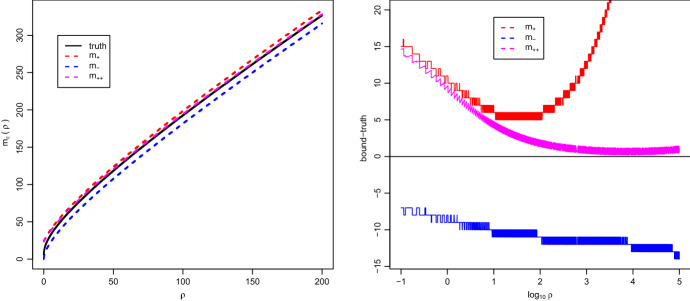
Left panel: $m_{\epsilon }(\rho )$ together with its upper and lower bounds from Theorem 1, plotted against $\rho$ for $\epsilon =10^{-16}$. Right panel $\mathsf {bound}(\rho )-m_{\epsilon }(\rho )$ against $\log _{10} \rho$ for $\epsilon =10^{-16}$

## A Evaluating $m_{\epsilon }(\rho )$

Our fast, robust and accurate method for evaluating $m_{\epsilon }(\rho )$, as defined in Sect. 3.1 relies on the following new result.

### Theorem 1

If $\rho \le \epsilon$, $m_{\epsilon }(\rho )=0$, and if $\rho \le \epsilon ^{1/2}$, $0\le m_{\epsilon }(\rho )\le 1$. More generally: $m_{\epsilon }(\rho )\le \lceil m_+\rceil$, where

8 $$\begin{aligned} m_+:= \rho -\frac{1}{3}\log \epsilon \left\{ 1+\left( 1-\frac{18\rho }{\log \epsilon }\right) ^{1/2}\right\} -1. \end{aligned}$$

Furthermore,

$$\begin{aligned} \lfloor m_-\rfloor \le m_{\epsilon }(\rho ) \le \lceil m_{++}\rceil , \end{aligned}$$

where both inequalities require $\epsilon <0.04$ and the latter also requires $\epsilon <1-e^{-\rho }$ and $B>\log \epsilon$, where

9 $$\begin{aligned}&m_-:=\rho +\{2\rho \}^{1/2}\left\{ -\log (\epsilon \sqrt{2\pi })-\frac{3}{2}\log A+\log (A-1)\right\} ^{1/2}, \end{aligned}$$

10 $$\begin{aligned}&\quad m_{++}:=\rho +\frac{B-\log \epsilon }{3}\left\{ 1+\left( 1+\frac{18\rho }{B-\log \epsilon }\right) ^{1/2}\right\} ,\nonumber \\&\quad A:=2\rho {\mathsf {h}}\left( \frac{m_+ +1}{\rho }\right) ~~~ \text{ and } ~~~ B:= -\frac{1}{2}\log 4\pi \rho {\mathsf {h}}\left( \frac{m_-}{\rho }\right) , \end{aligned}$$

and $${\mathsf {h}}(x)=x-1+x\log x$$.

The bound (8) arises from a standard argument, whereas those in (9) and (10) are derived from extremely sharp but intractable bounds on $r_m(\rho ):={\mathbb {P}} \left( {\mathsf {Poisson}(\rho )>m}\right)$ in Short (2013); our bounds use only elementary functions and so are much quicker to compute than the quantile upper bound in Short (2013), yet from Fig. 2 they are still very sharp. The bounds in (9) and (10) together with the alternative form in (11)

11 $$\begin{aligned} r_m(\rho )=\frac{1}{\Gamma (m+1)}\int _0^\rho x^{m}e^{-x}\text{ d }x, \end{aligned}$$

which follows from the equivalence between at least $m+1$ events of a Poisson process with a unit rate occuring by time $\rho$ and the time until the $m+1$th event being at most $\rho$, permit a simple but fast binary search for $m_{\epsilon }(\rho )$.

### A.1 Implementation details

Our binary search algorithm homes in on the required *m* using the upper and lower bounds of Theorem 1 together with the identity (11), the right hand side of which can be evaluated quickly and accurately using the standard C++ toolbox, boost. This is quicker than the standard implementation of the Poisson quantile function (e.g. as implemented in boost), which uses the Cornish-Fisher expansion to approximate the quantile (hence needing an expensive evaluation of $\Phi ^{-1}$) and then conducts a local search.

### A.2 Proof of Theorem 1

The simple bounds for small $\rho$ arise because $e^{-\rho }>1-\rho$. Hence $r_0(\rho )=1-e^{-\rho }<\rho$ and if $\rho \le \epsilon$, $m_{\epsilon }(\rho )=0$. Furthermore, $r_1(\rho )=1-e^{-\rho }(1+\rho )<\rho ^2$, so if $\rho \le \sqrt{\epsilon }$ then $r_1(\rho )\le \epsilon$, so $m_{\epsilon }(\rho )\le 1$.

The other bounds all use aspects of the following result.

#### Lemma 1

Let ${\mathsf {h}}(x):=1-x+x\log x$, then for $x\ge 1$,

$$\begin{aligned} \frac{3}{6+2(x-1)}(x-1)^2\le {\mathsf {h}}(x)\le \frac{1}{2}(x-1)^2. \end{aligned}$$

#### Proof

The left hand inequality holds for $x\ge 0$ and is from Boucheron et al. (2013) page 36. For the right hand inequality, set $g(x)=(x-1)^2/2$ and notice that $0={\mathsf {h}}(1)=g(1)={\mathsf {h}}'(1)=g'(1)$, and ${\mathsf {h}}''(x)=1/x\le 1=g''(x)$ for $x\ge 1$. $\square$

The first upper bound on $m_{\epsilon }(\rho )$, (8), arises from a standard Chernoff argument (e.g. Boucheron et al. (2013)) to the right tail of a $\mathsf {Poisson}(\rho )$ random variable, *X*. The moment generating function is $M_X(t)={\mathbb {E}} \left[ {e^{Xt}}\right] =\exp [\rho (e^t-1)]$, and by Markov’s inequality:

$$\begin{aligned} {\mathbb {P}} \left( {X\ge m}\right) ={\mathbb {P}} \left( {e^{Xt}\ge e^{mt}}\right) \le e^{-mt}M_{X}(t) =e^{-mt+\rho (e^t-1)}. \end{aligned}$$

The inequality holds for all *t* and the right-hand side is minimised at $t=\log (m/\rho )$, giving

$$\begin{aligned} {\mathbb {P}} \left( {X\ge m}\right) \le \exp [-\rho {\mathsf {h}}(m/\rho )]\le \exp \left[ -\rho \frac{3(m/\rho -1)^2}{6+2(m/\rho -1)}\right] \end{aligned}$$

by Lemma 1. Setting $\epsilon ={\mathbb {P}} \left( {X\ge m+1}\right)$ and $y=(m+1)/\rho -1$ gives $3\rho y^2 (6+2y)\log \epsilon \ge 0$, from which $y\ge -\log \epsilon \times \sqrt{1-18\rho /\log \epsilon }/(3\rho )$, and (8) follows on substituting for *y*.

The much tighter bounds in (9) and (10) use Theorem 2 of Short (2013), which can be rewritten to state that

12 $$\begin{aligned} \Phi \left( -\sqrt{2\rho {\mathsf {h}}(m'/\rho )}\right)< {\mathbb {P}} \left( {X> m}\right) < \Phi \left( -\sqrt{2\rho {\mathsf {h}}(m/\rho )}\right) , \end{aligned}$$

where $m':=m+1$ and where the left hand side holds provided $m'>\rho$ and the right hand side holds provided $m>\rho$. We first show that these conditions are satisfied. Firstly, when $\rho <1$, clearly $m'>\rho$, moreover $r_0(\rho )=1-e^{-\rho }$, so provided $1-e^{-\rho }>\epsilon$, we require $m\ge 1>\rho$. When $\rho \ge 1$, we use the easily verified facts that $r_{m}(m)$ is an increasing function of *m* and $r_{m}(\rho )$ is an increasing function of $\rho$; thus for $\rho \ge m \ge 1$, $r_m(\rho )\ge r_{m}(m)\ge r_1(1)=1-2e^{-1}>0.04$, and the tolerance condition is not satisfied. We, therefore need $m>\rho$ (which also gives $m'>\rho$).

Neither $\Phi ^{-1}$ nor ${\mathsf {h}}^{-1}$ is tractable (functions that perform $\Phi ^{-1}(p)$ solve $\Phi (x)=p$ iteratively), and even with the bounds on ${\mathsf {h}}$ from Lemma 1 and standard bounds on $\Phi$ in terms of $\phi$, tractable inversion is still not possible. We use the bound (8) to create (9), and then (9) to create (10).

To prove (9), since $\epsilon \le 0.04$, from the left inequality in (12),

$$\begin{aligned} 0.04 \ge {\mathbb {P}} \left( {X\ge m}\right) \Rightarrow \sqrt{2\rho {\mathsf {h}}(m'/\rho )}\ge -\Phi ^{-1}(\epsilon )\approx 1.75> \sqrt{3}. \end{aligned}$$

Firstly, since $m_++1\ge m+1$, this ensures $A>1$, so $\log (A-1)$ is real. More importantly, it ensures that $[2\rho {\mathsf {h}}(m'/\rho )]^{-1/2}-[2\rho {\mathsf {h}}(m'/\rho )]^{-3/2}$ is a decreasing function of $[2\rho {\mathsf {h}}(m'/\rho )]^{1/2}$ and, since ${\mathsf {h}}'(x)>0$ for $x>1$, it is also a decreasing function of $m'$. The $m'$ that we desire satisfies $m'\le m_++1=:m_+'$, and hence

$$\begin{aligned}{}[2\rho h(m'/\rho )]^{-1/2}-[2\rho h(m'/\rho )]^{-3/2} \ge [2\rho h(m_+'/\rho )]^{-1/2}-[2\rho h(m_+'/\rho )]^{-3/2}. \end{aligned}$$

Since, for $$y>0$$, $$\Phi (-y)>(1/y-1/y^3) \phi (y)$$,

$$\begin{aligned} \Phi \left( -\sqrt{2\rho h(m'/\rho )}\right) \ge \left\{ [2\rho h(m_+'/\rho )]^{-1/2}-[2\rho h(m_+'/\rho )]^{-3/2}\right\} \phi \left( \sqrt{2\rho h(m'/\rho )}\right) . \end{aligned}$$

Combining the left inequality in (12) with the right-hand inequality in Lemma 1 gives

$$\begin{aligned} \epsilon \ge \frac{1}{\sqrt{2\pi }} \left\{ [2\rho h(m_+'/\rho )]^{-1/2}-[2\rho h(m_+'/\rho )]^{-3/2}\right\} \exp \left[ -\frac{(m'-\rho )^2}{2\rho }\right] . \end{aligned}$$

Equation (9) follows on rearrangement.

To show (10) we apply the right hand inequality in (12) and the bound $\Phi (-x)< \phi (x)/x$, then the fact that $m\ge m_-$, and finally Lemma 1 to find:

$$\begin{aligned} {\mathbb {P}} \left( {X> m}\right)&< \frac{1}{\{4\pi \rho {\mathsf {h}}(m/\rho )\}^{1/2}} \exp \left[ -\rho {\mathsf {h}}(m/\rho )\right] \\&\le \frac{1}{\{4\pi \rho {\mathsf {h}}(m_-/\rho )\}^{1/2}} \exp \left[ -\rho {\mathsf {h}}(m/\rho )\right] \\&\le \frac{1}{\{4\pi \rho {\mathsf {h}}(m_-/\rho )\}^{1/2}} \exp \left[ -3\rho \frac{(x-1)^2}{6+2(x-1)}\right] , \end{aligned}$$

where $x=m/\rho$. We must, therefore, ensure that the final bound is no more than $\epsilon$. Rearranging this gives $3\rho (x-1)^2-2(B-\log \epsilon )(x-1)-6(B-\log \epsilon )\le 0$, so that when $B-\log \epsilon >0$, $x-1\le (B-\log \epsilon )(1+\sqrt{1+18\rho /(B-\epsilon )})/(3\rho )$.

## B Scaling and squaring: choosing *s*

Recall that the scaling and squaring algorithm evaluates $\exp ({\mathsf {M}})$ via the equality $\exp ({\mathsf {M}})=\left[ \exp ({\mathsf {M}}/2^s)\right] ^{2^s}$. Calculation of $\exp ({\mathsf {M}}/2^s)$ takes $m_{\epsilon }(\rho /2^s)$ matrix-matrix multiplications; repeatedly squaring this quantity *s* times takes *s* matrix-matrix multiplications, leading to a total cost of

$$\begin{aligned} c(s)=m_{\epsilon }(\rho /2^s)+s. \end{aligned}$$

Asymptotically in large $\rho$, $\mathsf {Pois}(\rho )/\rho \sim 1$, so $m_{\epsilon }(\rho )\approx \rho$. With $m_{\epsilon }(\rho /2^s)=\rho /2^s$, *c*(*s*) is minimised at

$$\begin{aligned} {\widehat{s}}_1= \frac{\log \rho +\log \log 2}{\log 2}. \end{aligned}$$

Since $m_{\epsilon }(\rho )$ is an upper quantile, we also have that $m_{\epsilon }(\rho )> \rho$, and, for low $\rho$, we find $m_{\epsilon }(\rho )\gg \rho$. Indeed, $m_{\epsilon }(\rho /2^s)$ does not, in fact, decrease as quickly as $\rho /2^s$, so

$$\begin{aligned} c'(s)=\frac{\text{ d }}{\text{ d }s}m_{\epsilon }(\rho /2^s) +1 \ge \frac{\text{ d }}{\text{ d }s} \frac{\rho }{2^s}+1. \end{aligned}$$

Since the gradients both become less negative as *s* increases (indeed, they asymptote to 0), ${\widehat{s}}_1$ is a strict lower bound on the true minimum ${\widehat{s}}$. For a range of $\epsilon$ values from 0.1 to $10^{-16}$, and a range of $\rho$ values from 0.1 to $10^6$, we have found that the true optimum *s* always lies in the range between ${\widehat{s}}_1$ and ${\widehat{s}}_1+6$. When the scaling and squaring algorithm is required, ${\widehat{s}}$ chosen as the integer argument within this range that minimises *c*(*s*).

When ${\mathsf {M}}$ is dense, the above algorithm finds the optimal choice of *s*; however, when ${\mathsf {M}}$ is sparse, the matrix multiplications required to evaluate $\exp ({\mathsf {M}}/2^s)$ are cheaper than those involved in the subsequent squaring. Hence, the cost is minimised at a slightly lower *s*, which depends on the sparsity of ${\mathsf {M}}$ (as well as $\rho$ and $\epsilon$). For simplicity, we set ${\widehat{s}}\leftarrow {\widehat{s}}-\min (2,{\widehat{s}})$.

## C Functions in the expQ package

The functions in the expQ package are provided below. Each function requires a rate matrix, *Q*, which can be sparse or dense, and a precision, $\epsilon$. Unif_v_exp_Q takes a horizontal vector, *v*, and calculates $v\exp {\mathsf {Q}}$ via uniformisation. SS_v_exp_Q takes a horizontal vector, *v*, and calculates $v\exp {\mathsf {Q}}$ via scaling and squaring. v_exp_Q takes a horizontal vector, *v*, and calculates $v\exp {\mathsf {Q}}$ via whichever is likely (based on empirical results on an i7-3770 CPU) to be the more efficient of uniformisation or scaling and squaring. vT_exp_Q takes a vertical vector, *v*, and calculates $\left( v^\top \exp {\mathsf {Q}}\right) ^\top$ via whichever is likely (based on empirical results on an i7-3770 CPU) to be the more efficient of uniformisation or scaling and squaring. SS_exp_Q calculates $\exp {\mathsf {Q}}$ using scaling and squaring.

## D Latent-variable updates for the SIR model

Our particular reduced-statespace implementation of the SIR model fit for the Swansea Measles epidemic uses 10 additional latent observation times, 5 between days 120 and 151 (at days 125, 130, 135, 140 and 145) and five between days 151 and 212 (at days 155, 160, 165, 170 and 175). This leads to 27 latent variables: 17 unknown number of infecteds at the (true and latent) observation times and 10 (not 12 because two sums are known) unknown numbers of recovered for the time period since the previous (true or latent) observation time. We emphasise that the *R* latent variables are *not* the cumulative number of recovered individuals since the epidemic began.

When a new latent vector is proposed, we first check whether it can possibly fit with the current $n_{pop}$ and the known data. If it does not fit, then the proposal may be rejected without any matrix exponentiation. At the *j*th (true or latent) time point, denote the current number of infecteds by $I_j$ and the number removed since the previous time point by $R_j$. Let *J* be the total number of (true and latent) time points. Note that $S_j=n_{pop}-I_0-I_j-\sum _{i=1}^j R_i$. The following checks are performed:

1. For each $j=1,\dots ,J$: $I_j\ge 0$, $R_j \ge 0$ and $S_j\ge 0$.
2. For each $$j=1,\dots ,J$$: $$S_{j}\le S_{j-1}$$.
3. For each $$j=1,\dots ,J-1$$: $$I_j\le \sum _{i=j+1}^JR_i$$.

The third constraint arises because there are no active infections at the end of the epidemic. These constraints can hinder the mixing of the integer-valued random walk algorithm on the latent variables, so we split the latent variables into four groups, grouped by observation time. This grouping has the additional advantage that only a subset of matrix exponentiation calculations need be performed for each of the four individual proposals.

## E Log-likelihood R code for the Moran model

To demonstrate the simplicity of inference via the matrix exponential, we provide code to evaluate the log-likelihood for the Moran model. Code for the filtering distribution is very similar but there is no need to track the re-normalisation constant (in $\texttt {ll}$).
